# A sensorimotor enhanced neuromusculoskeletal model for simulating postural control of upright standing

**DOI:** 10.3389/fnins.2024.1393749

**Published:** 2024-05-15

**Authors:** Julian Shanbhag, Sophie Fleischmann, Iris Wechsler, Heiko Gassner, Jürgen Winkler, Bjoern M. Eskofier, Anne D. Koelewijn, Sandro Wartzack, Jörg Miehling

**Affiliations:** ^1^Engineering Design, Department of Mechanical Engineering, Friedrich-Alexander-Universität Erlangen-Nürnberg, Erlangen, Germany; ^2^Machine Learning and Data Analytics Lab, Department Artificial Intelligence in Biomedical Engineering (AIBE), Friedrich-Alexander-Universität Erlangen-Nürnberg, Erlangen, Germany; ^3^Department of Molecular Neurology, Universitätsklinikum Erlangen, Friedrich-Alexander-Universität Erlangen-Nürnberg, Erlangen, Germany; ^4^Chair of Autonomous Systems and Mechatronics, Department of Electrical Engineering, Friedrich-Alexander-Universität Erlangen-Nürnberg, Erlangen, Germany

**Keywords:** postural control, standing, simulation, forward dynamics, neuromusculoskeletal modeling, neural control, motor control, biomechanics

## Abstract

The human's upright standing is a complex control process that is not yet fully understood. Postural control models can provide insights into the body's internal control processes of balance behavior. Using physiologically plausible models can also help explaining pathophysiological motion behavior. In this paper, we introduce a neuromusculoskeletal postural control model using sensor feedback consisting of somatosensory, vestibular and visual information. The sagittal plane model was restricted to effectively six degrees of freedom and consisted of nine muscles per leg. Physiologically plausible neural delays were considered for balance control. We applied forward dynamic simulations and a single shooting approach to generate healthy reactive balance behavior during quiet and perturbed upright standing. Control parameters were optimized to minimize muscle effort. We showed that our model is capable of fulfilling the applied tasks successfully. We observed joint angles and ranges of motion in physiologically plausible ranges and comparable to experimental data. This model represents the starting point for subsequent simulations of pathophysiological postural control behavior.

## 1 Introduction

Human upright standing is inherently unstable. Nevertheless, the human's postural control system is able to produce muscle forces and thereby joint torques to maintain the body in an upright position, even against external perturbations. Up to a certain degree, this is possible without the need to take a correction step. One typical symptom of neurological disorders like Parkinson's disease (PD) is an impaired function of the postural control system which results in difficulties maintaining balance during daily tasks. A detailed understanding of the body's internal control processes during postural control is essential to explain pathophysiological postural control and to be able to give tailored therapy recommendations to patients suffering from neurological disorders like PD.

To maintain balance, the human body continuously initiates muscle forces to keep the center of mass (COM) within the base of support (Winter, 1995). The base of support is defined by the area beneath the contact points of the feet with the ground. The central nervous system regulates information from the somatosensory, vestibular and visual systems to gain current body states and initiates suitable muscle excitations that lead to adequate muscle forces to keep the body in balance (Forbes et al., 2018). The somatosensory system consists of proprioception and cutaneous receptors. Proprioceptive information is perceived by muscle spindles and Golgi tendon organs. Muscle spindles are located in the skeletal muscles and sense muscle lengths and lengthening velocities (Kröger and Watkins, 2021). Golgi tendon organs are located at the interface between muscle and tendon and sense muscle tendon forces. Cutaneous receptors deliver tactile information about the pressure distribution underneath the feet (Jahn and Wühr, 2020), which includes changes in the location of the center of pressure (COP). The vestibular system is sensitive to linear and angular motion and orientation of the head. It consists of two structures located within the inner ear, the otolith organs and semicircular canals. Otolith organs detect linear accelerations as well as the head tilt with respect to the gravitational field, semicircular canals the rotational head accelerations (Mahboobin et al., 2002; Jahn and Wühr, 2020). The visual system provides information about the direction and speed of body sway (Jahn and Wühr, 2020). All these sensory information are centrally integrated to ensure a reliable and robust interpretation of the body state that can be used for postural control reactions. Lower level controls, like reflexes, are generated in the spinal cord, higher-level controls in the supra-spinal cord (Jahn and Wühr, 2020). This process is subject to neural delays consisting of processing, transmission and activation dynamics delays.

Biomechanical models can be used to simulate and analyze postural control behavior. Upright standing is often investigated using simplified models such as single inverted pendulum models (Masani et al., 2006; Welch and Ting, 2008; Goodworth and Peterka, 2009). More detailed models can consist of a higher number of degrees of freedom (DOF) and muscles (Versteeg et al., 2016; Kaminishi et al., 2019; Koelewijn and Ijspeert, 2020). Simulations of musculoskeletal human models often focus on proprioceptive information (Suzuki and Geyer, 2018; Koelewijn and Ijspeert, 2020) or assume the body's full-state information to be known by the central nervous system (Welch and Ting, 2008; Yin et al., 2020). Also, the considered amount of neural delays varies a lot between the different models. An overview about different simulation approaches of postural control, which biomechanical human models and control strategies are used, is given by Shanbhag et al. (2023). To gain a detailed understanding about internal processes during postural control, it is necessary to consider all of the different sensory systems that the human body uses to maintain balance. A clear distinction between the origins of different sensory signals used by a model, like proposed by Jiang et al. (2017), is rarely done in postural control simulations. However, detailed postural control models considering such distinctions and all sensory systems used for postural control, and also neural delays in physiologically plausible ranges, could be capable of covering many aspects of postural control and giving insights into internal processes of the body that are still not fully understood.

In this paper, we use a forward dynamic approach to simulate balance control. We introduce a postural control model for upright standing using a musculoskeletal human model with nine DOF and 18 muscles. The model considers somatosensory, vestibular as well as visual information for generating muscle feedback. Therefore, this feedback consists of reflexes based on muscle information, enhanced by additional information gained from cutaneous sensors, the vestibular and visual system. Also, physiologically plausible neural delays are added within the neural circuitry, depending on muscle position and information type. The model is able to simulate quiet and perturbed upright standing.

## 2 Materials and methods

We used a generic musculoskeletal human model to conduct forward-dynamic simulations of postural control behavior of quiet and perturbed upright standing. The simulations in this study were applied using the software framework SCONE 2.3.0 (Geijtenbeek, 2019) with Hyfydy (Geijtenbeek, 2021). The implementation consists of three elements, which is described in the following: A musculoskeletal human model, a neural controller and an optimization of free control parameters. Additionally, the simulation approach and the experimental data with which the simulation results are compared are described.

### 2.1 Musculoskeletal model

For our simulations, we used a musculoskeletal human model based on Delp et al. (1990) with updates from Rajagopal et al. (2016), distributed as part of SCONE. This version is a planar model (sagittal plane) with seven segments, consisting of trunk-pelvis, and each upper leg, lower leg and foot. The model is restricted to nine DOF, three DOF per leg (ankle, knee and hip joint) and three DOF between the pelvis and the ground. In our simulations, we assumed a symmetric motion behavior as we wanted to show a general physiological motion behavior. As a result, the model effectively has six DOF, left and right joint angles were treated identically. Nine Hill-type muscles (Millard et al., 2013) are considered for each leg: Gluteus maximus (GLU), hamstrings (HAM), iliopsoas (IL), rectus femoris (RECT), biceps femoris short head (BFSH), vastus intermedius (VAS), gastrocnemius medialis (GAS), soleus (SOL), and tibialis anterior (TA). Values for muscle parameters were set according to Delp et al. (1990) with updates from Rajagopal et al. (2016). Ground contact is modeled via two viscoelastic Hunt-Crossley contact spheres per foot, one on the heel and one on the forefoot.

### 2.2 Neural control design

**Figure 1:**
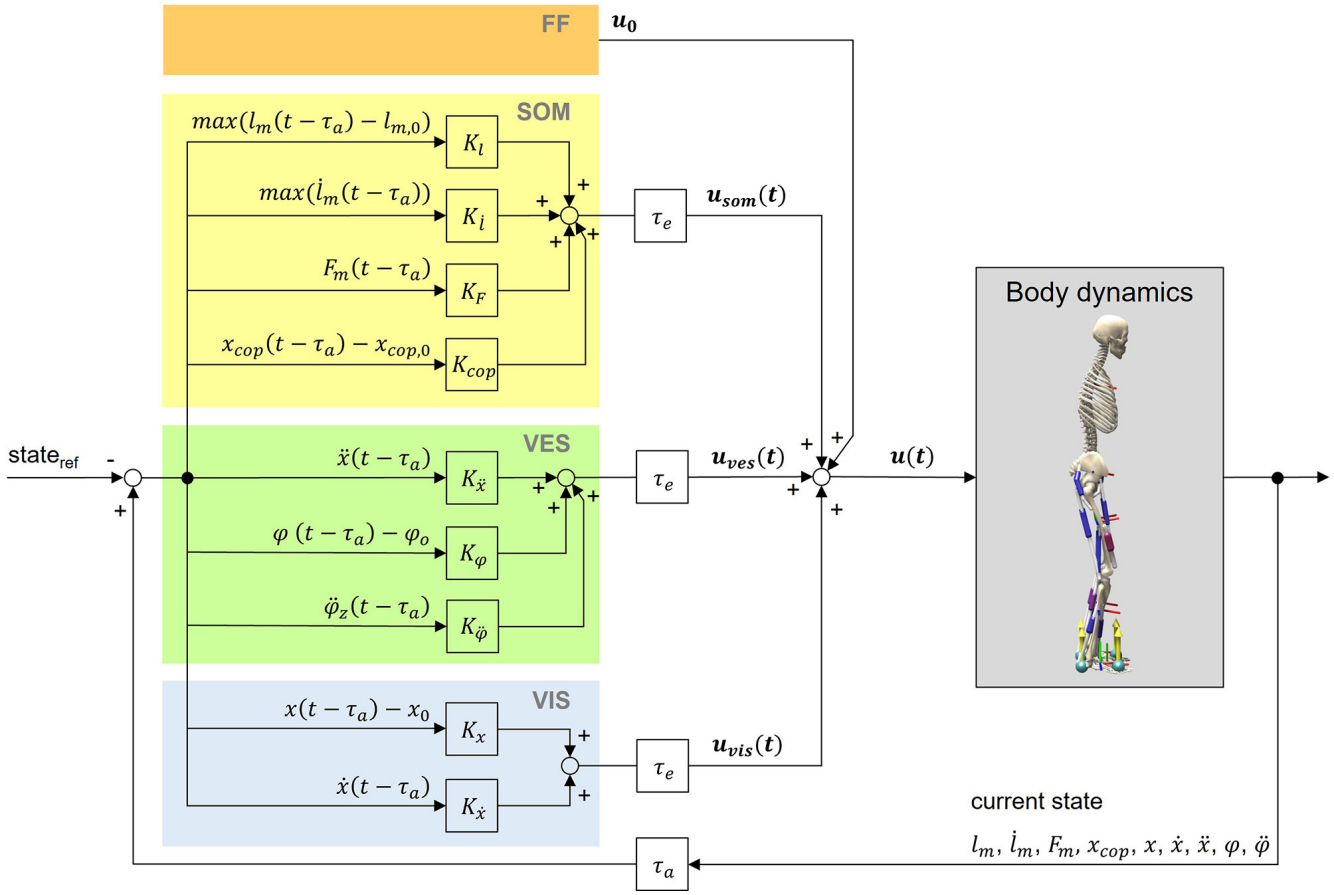
Postural control model. The neural controller generates control signals based on feedforward input (FF) as well as somatosensory (SOM), vestibular (VES) and visual (VIS) feedback. Feedback values are generated by comparing time-delayed states of the model with their corresponding reference states. Indicated delays belong to the body's afferences (τ_*a*_) and efferences (τ_*e*_). For calculations, we used a lumped delay τ consisting of both signal transmission and processing delays (Section 2.2.2). The reference position is an upright standing pose. Signals from the neural controller lead to muscle excitations *u*(*t*) of the musculoskeletal human model.

We implemented a neural control circuit to simulate postural control behavior of upright standing using the aforementioned musculoskeletal human model. Our aim was to establish processes on a physiologically plausible basis: All sensory information that are used in the physiological process of postural control should be available for the model's control as well. State information of the body, gained by the different sensory systems of the body, were considered to generate feedback signals, depending on their corresponding gain factors. Additionally, the feedback loop is subject to neural delays τ (Section 2.2.2). The whole postural control model is shown in Figure 1.

#### 2.2.1 Feedback controller

The total muscle excitation that is continuously calculated for each individual muscle is the sum of the different sensory systems' feedback and is represented in Equation (1):


(1) $$\begin{array}{l} u\left(t\right)=u_{0}+u_{som}\left(t\right)+u_{ves}\left(t\right)+u_{vis}\left(t\right) \end{array}$$

The total muscle excitation *u*(*t*) consists of a feedforward element *u*_0_, somatosensory feedback *u*_*som*_(*t*), vestibular feedback *u*_*ves*_(*t*) and visual feedback *u*_*vis*_(*t*). Equations (2–4) are used to determine the corresponding feedback elements.


(2) $$\begin{array}{l} \begin{array}{c} u_{som}\left(t\right)=K_{l}\cdot max\left(0,\left(l_{m}\left(t-\tau \right)-l_{m,0}\right)\right)+K_{\dot{l}}\cdot max\left(0,{\dot{l}}_{m}\left(t-\tau \right)\right)\\ +K_{F}\cdot F_{m}\left(t-\tau \right)+K_{cop}\cdot \left(x_{cop}\left(t-\tau \right)-x_{cop,0}\right) \end{array} \end{array}$$

*K*_*l*_, $K_{\overset{•}{l}}$, *K*_*F*_, and *K*_*cop*_ are gain factors based on proprioceptive and tactile information of the somatosensory system. *l*_*m*_(*t*−τ), ${\overset{•}{l}}_{m}\left(t-\tau \right)$, and *F*_*m*_(*t*−τ) represent the time-delayed normalized length, lengthening velocity and force of the corresponding muscle. *l*_*m*, 0_ is the offset length of the muscle above which the muscle initiates length feedback. *x*_*cop*, 0_ is the initial COP value and the midpoint between the two contact points of the feet with the ground. It was assumed that if the projection of the center of mass and the COP coincide in this point, the model reaches an equilibrium posture.


(3) $$u_{ves}(t)=K_{\ddot{x}}\cdot \ddot{x}(t-\tau )+K_{\varphi }\cdot (\varphi (t-\tau )-\varphi_{0})+K_{\ddot{\varphi }}\cdot \ddot{\varphi }(t-\tau )$$

*K*_ẍ_, *K*_φ_, and $K_{\ddot{\varphi }}$ are gain factors based on vestibular information. ẍ(*t*−τ) represents the time-delayed linear acceleration, φ(*t*−τ) the time-delayed orientation and $\ddot{\varphi }\left(t-\tau \right)$ the time-delayed angular acceleration of the head with respect to the environment. φ_0_ is the initial orientation of the head.


(4) $$u_{vis}(t)=K_{x}\cdot (x(t-\tau )-x_{0})+K_{\dot{x}}\cdot \dot{x}(t-\tau )$$

*K*_*x*_ and *K*_ẋ_ are gain factors based on visual information. *x*(*t*−τ) and ẋ(*t*−τ) represent the time-delayed position and velocity of the head with respect to the environment.

All feedback gains, the offset muscle lengths *l*_*m*, 0_ as well as feedforward excitations *u*_0_ were optimized in the optimization step (Section 2.3). The calculated muscle excitations *u*(*t*) lead to muscle activations and subsequently adjusted muscle lengths, lengthening velocities and forces that are determined via the activation and contraction dynamics of the Hill-type muscle model (Millard et al., 2013).

Additionally, we applied a small amount of random Gaussian noise to the model's sensor systems as well as to the actuators assuming that these systems do not work noise free in the human body, consisting of base noise and noise depending on the signal amplitude *s*, which is either sensor information or muscle excitation (Equations 5, 6):


(5) $$\begin{array}{ll} x^{\prime } & =x+k_{noise}\cdot R \end{array}$$

(6) $$\begin{array}{ll} k_{noise} & =0.0005+0.0001\cdot s \end{array}$$

*x* is the true signal, *k*_*noise*_ the signal-dependent noise amplitude and *R* a randomly generated Gaussian distributed number. *x*′ represents the resulting signal's value.

#### 2.2.2 Neural delays

Depending on the muscle position in the body and the sensory information type, we considered different amounts of neural delays. For each muscle we defined a lumped neural delay τ consisting of transmission and processing delay. We set neural delays according to assumptions of Li et al. (2012). For muscle reflexes based on proprioceptive information reactions up to 25 ms are possible. Depending on the muscle's position, the reflex delay can increase by additional 25 ms due to the longer transmission distance from the central nervous system to shank muscles compared to hip muscles. For vestibular and visual information a higher amount of processing is necessary. Therefore, additional 100 ms were assumed for each muscle. All neural delays that we used are summarized in Table 1.

**Table 1:** Neural delays for each muscle depending on the muscle's position and the sensory information type.

**Muscle**	**Neural delays**		
	**Somatosensory information**	**Vestibular information**	**Visual information**
Gluteus maximus, hamstrings, iliopsoas, rectus femoris	25 ms	125 ms	125 ms
Biceps femoris short head, vastus intermedius	35 ms	135 ms	135 ms
Gastrocnemius medialis, soleus, tibialis anterior	50 ms	150 ms	150 ms

### 2.3 Optimization of control parameters

The control gains, offset muscle lengths and feedforward excitations were optimized using single shooting and the pre-implemented covariance matrix adaption evolution strategy (CMA-ES) algorithm (Igel et al., 2007) in SCONE. An optimization was solved to find control parameters to minimize muscular effort and to fulfill additional constraints, such as knee and hip joint limits and keeping the model's COM higher than 60% of the initial height. Effort minimization is chosen as this is assumed to be the objective of the central nervous system when creating movements (Selinger et al., 2015). This leads to the following optimization problem (Equations 7–11):


(7) $$\begin{array}{l} J_{\text{cost}}=100\cdot J_{\text{fall}}+0.01\cdot J_{\text{effort}}+10\cdot J_{\text{knee joint}}+0.1\cdot J_{\text{hip joint}} \end{array}$$

(8) $$J_{\text{fall}}=\{\begin{array}{ll} 1, & \text{if COM height}<\text{0}.\text{6 }\cdot \text{ initial COM height}\\ 0, & \text{else} \end{array}$$

(9) $$J_{\text{effort}}=\frac{1}{T}\int_{t=0}^{T}\sum_{i=1}^{N_{musc}}a_{i}^{3}dt$$

(10) $$J_{\text{knee joint}}=\{\begin{array}{ll} 0, & \text{if }-30^{{}^{\circ}}<\text{knee angle}<0^{{}^{\circ}}\\ 1, & \text{else} \end{array}$$

(11) $$J_{\text{hip joint}}=\{\begin{array}{ll} 0, & \text{if }-20^{{}^{\circ}}<\text{hip flexion}<40^{{}^{\circ}}\\ 1, & \text{else} \end{array}$$

*N*_*musc*_ represents the number of muscles and *a*_*i*_ the activation of each muscle. For each scenario (described in Section 2.4), six optimizations with different random seeds were performed. During this process, multiple CMA-ES optimizations are carried out and prioritized depending on their predicted fitness values (Geijtenbeek, 2019). Optimizations ended as soon as the averaged reduction of the cost function's result was smaller than 1e-5 compared to the previous iteration.

In this model, we assumed a symmetric postural control behavior, so left and right muscle excitations are calculated identically. For parameters' initial guesses, we considered findings of Peterka (2018) where sensory feedback weightings were identified. They reported postural control to be based 50% on proprioceptive, 33% on visual, and 17% on vestibular information for low amplitude perturbations. We considered this ratio for our feedback gains' initial guesses, where applicable. Boundaries for parameter optimization were defined as –3 and 3 for control gains, as 0.001 and 0.2 for feedforward excitations *u*_0_, and as 0.1 and 2 for the muscle's offset lengths *l*_*m*, 0_. Boundaries for control gains were adopted from Koelewijn and Ijspeert (2020), for feedforward excitations and offset lengths they were adapted to reduce the search space.

### 2.4 Simulation approach

Full simulations lasted 75 s. We evaluated simulation results for 60 s in total, starting from 15 s simulation time, because the first seconds are not representative due to a manually predefined starting position. Simulation frequency was set to 200 Hz.

The model's initial posture was always an upright stance (pelvis tilt: −10.0°, hip flexion: 20.0°, knee angle: –20°, ankle angle: 10°) which was adopted from the pre-defined SCONE settings for standing simulations (Geijtenbeek, 2019). For evaluation, we tested our postural control model under two scenarios: In the first step, we simulated a quiet standing (no external perturbations). Results were compared to self-collected experimental data. Additionally, we tested our model performing an upright standing task on a moving platform. We applied translational perturbations to the platform comparable to the study of Wang and van den Bogert (2020). This resulted in anterior-posterior perturbations of the model and allowed us to compare our simulation results to the experimental results of this open accessible dataset. In their study, a random square signal was applied to the platform. The signal consisted of different amplitudes of [–5, –2.5, 0, 2.5, 5] cm, and six stage durations of [0.25, 0.5, 0.75, 1.0, 1.25, 1.5] s. Platform velocities of up to ~0.22 m/s were observed.

### 2.5 Experimental data

In this paper, we used two different dataset to compare our simulation results to experimental data. For a quiet upright standing, we conducted own data, for a perturbed upright standing on a moving platform, we used an open-source dataset from Wang and van den Bogert (2020).

We conducted a study to measure postural control behavior during quiet upright standing. Eight healthy participants (four male, four female, age: 51.63 ± 23.12 years) were included in this study. All participants performed an upright standing task with eyes open for 60 s. They were instructed to place their feet shoulder-width apart and focus on a sign at eye level in front of them. Hands were placed on the hips during this task. We collected the data in a motion laboratory using an optical motion capture system (VICON Vero, ten cameras, 100 Hz) and two force plates (AMTI, 1,000 Hz). We recorded the data with 45 reflective body markers according to the Plug-in Gait model (Vicon Motion Systems Limited UK, 2021), with four additional markers on each medial knee and ankle. These four medial markers were detached after an initial calibration measurement. For processing the experimental data, we used OpenSim 4.4 (Seth et al., 2018) and a three-dimensional model, based on Delp et al. (1990). The model consisted of 17 DOF and eight segments. The processing itself was conducted using the tool AddBiomechanics (Werling et al., 2023) that includes an automatic model scaling and an inverse kinematics processing from human motion data. In the end, results were filtered with a third order Butterworth filter with a cutoff frequency of 6 Hz.

Experimental data that we used to compare an upright standing on a moving platform was collected from six participants (Wang and van den Bogert, 2020). Each of them fulfilled two perturbed standing tasks. They collected optical motion capture, force plate and EMG data. Measurement data were processed using a two-dimensional sagittal plane model (three DOF) consisting of hip, knee and ankle joint. During calculations of this dataset, left and right joint angles were averaged, as movements were assumed to be symmetrical.

### 2.6 Data evaluation

To evaluate our postural control model for upright standing, we compared simulation results with experimental data (Section 2.5). We used own collected experimental data to evaluate the unperturbed scenario. For the upright standing on a moving platform, we compared simulation results with the publicly available dataset of Wang and van den Bogert (2020). In the dataset records, platform movement started after ~12 s. For evaluation, we used the time period between 15 and 75 s for both our simulation and experimental data to compare areas with a high amount of perturbation. For both scenarios we analyzed joint angles and COP values over 60 s. Additionally, for the upright standing on a moving platform, we compared curve progressions of experimental EMG data and simulated muscle activations, where applicable. Quantitative comparisons are not possible, since EMG data give no insights about absolute values of muscle activations.

We averaged experimental data to compare them with simulation results. We calculated RMSEs of simulations and experimental data as well as ROMs of simulations and average ROMs of experimental data. To calculate RMSEs, we used experimental data of each participant averaged for each time step resulting in average time courses. To gain average ROMs, we averaged all participant's individual ROMs. Because the absolute COP position depends on the definitions of the force plates' coordinate systems and standing positions of participants, we compared just the COP ranges. To gain COP results, COP data were obtained directly from the force plates. The two COP values from both of the force plates were fused to one resulting COP for each time step by determining a weighted sum. In this paper, we considered the anterior-posterior components for further analysis. To compare EMG data with simulation results for the perturbed upright standing, we pre-processed raw EMG data from Wang and van den Bogert (2020). First, we band-pass filtered the signals between 20 and 500 Hz to remove electrical noise and motion artifacts (McManus et al., 2020). Then, we rectified and lowpass filtered the signals by 6 Hz.

## 3 Results

**Figure 2:**
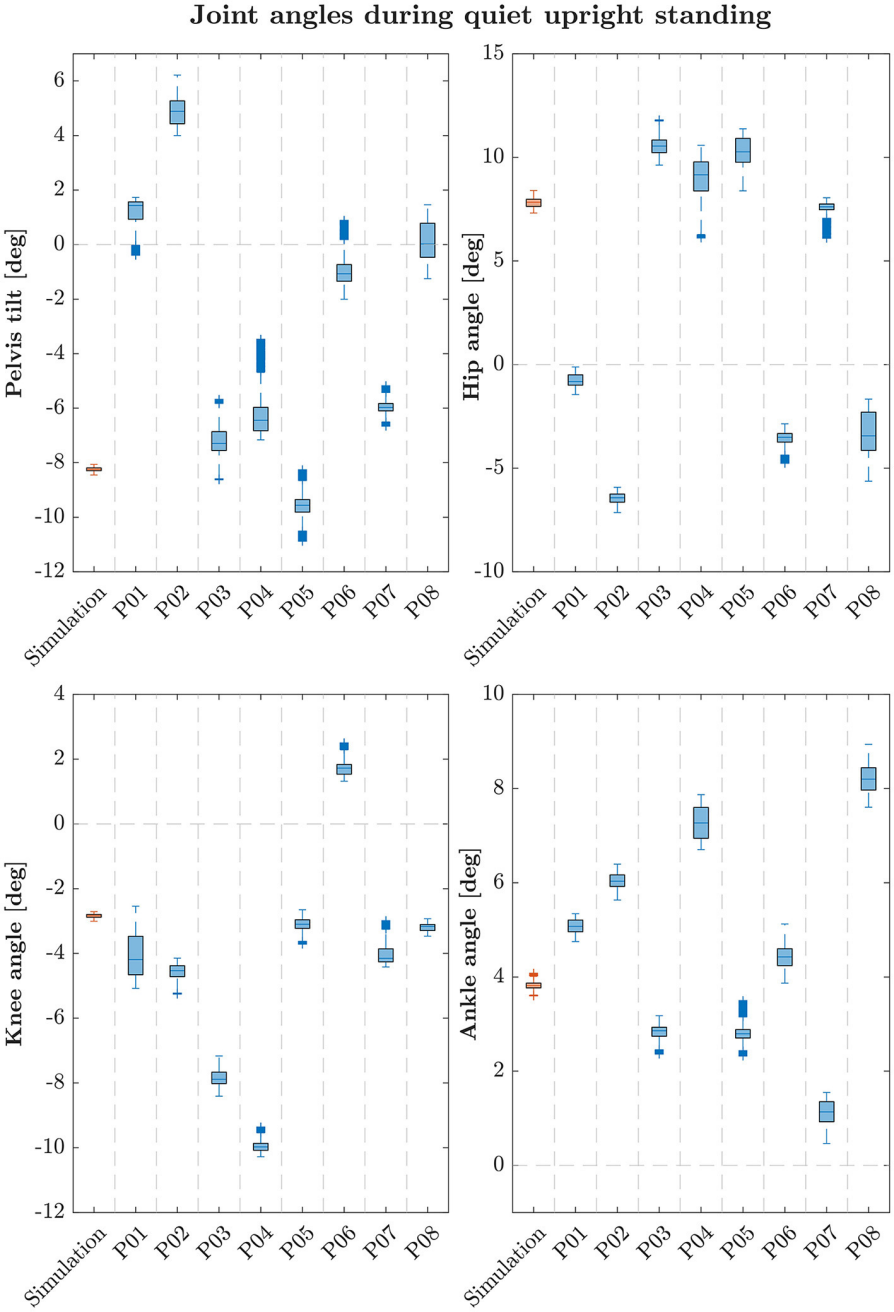
Box-plots showing median and interquartile ranges of joint angles during quiet upright standing of simulation and each participant. Simulations assumed left and right symmetry, experimental data were averaged for left and right joint angles of each participant following this assumption.

The simulation model was able to successfully fulfill the unperturbed standing task for the complete simulation time. Figure 2 shows joint angles of the simulation and each participant of the experiments. We observed RMSEs of 5.28° (pelvis tilt), 4.93° (hip angle), 1.54° (knee angle) and 0.91° (ankle angle) between simulations and average experimental data. Simulation results show joint angles' ROMs of 0.63° (pelvis tilt), 0.72° (hip angle), 1.16° (knee angle), and 0.67° (ankle angle). Average experimental data show ROMs of 2.59 ± 0.66° (pelvis tilt), 2.52 ± 1.16° (hip angle), 1.38 ± 0.54° (knee angle), and 1.11 ± 0.30° (ankle angle). The COP range was 11.96 mm during the simulation, 25.62 ± 8.65 mm during experiments. Table 2 summarizes RMSEs and ROMs of joint angles and the COP of each simulation and average experimental data.

**Table 2:** Joint angles' and COP's parameters of the simulation and average experimental data during quiet upright standing.

**Parameters**	**range of motion (ROM) simulation**	**ROM experiments**	**root mean square error (RMSE)**
Pelvis angle (deg)	0.63	2.59 ± 0.66	5.28
Hip angle (deg)	0.72	2.50 ± 1.20	4.93
Knee angle (deg)	1.16	1.25 ± 0.58	1.54
Ankle angle (deg)	0.67	1.02 ± 0.27	0.91
COP (mm)	11.96	25.62 ± 8.65	N/A

**Figure 3:**
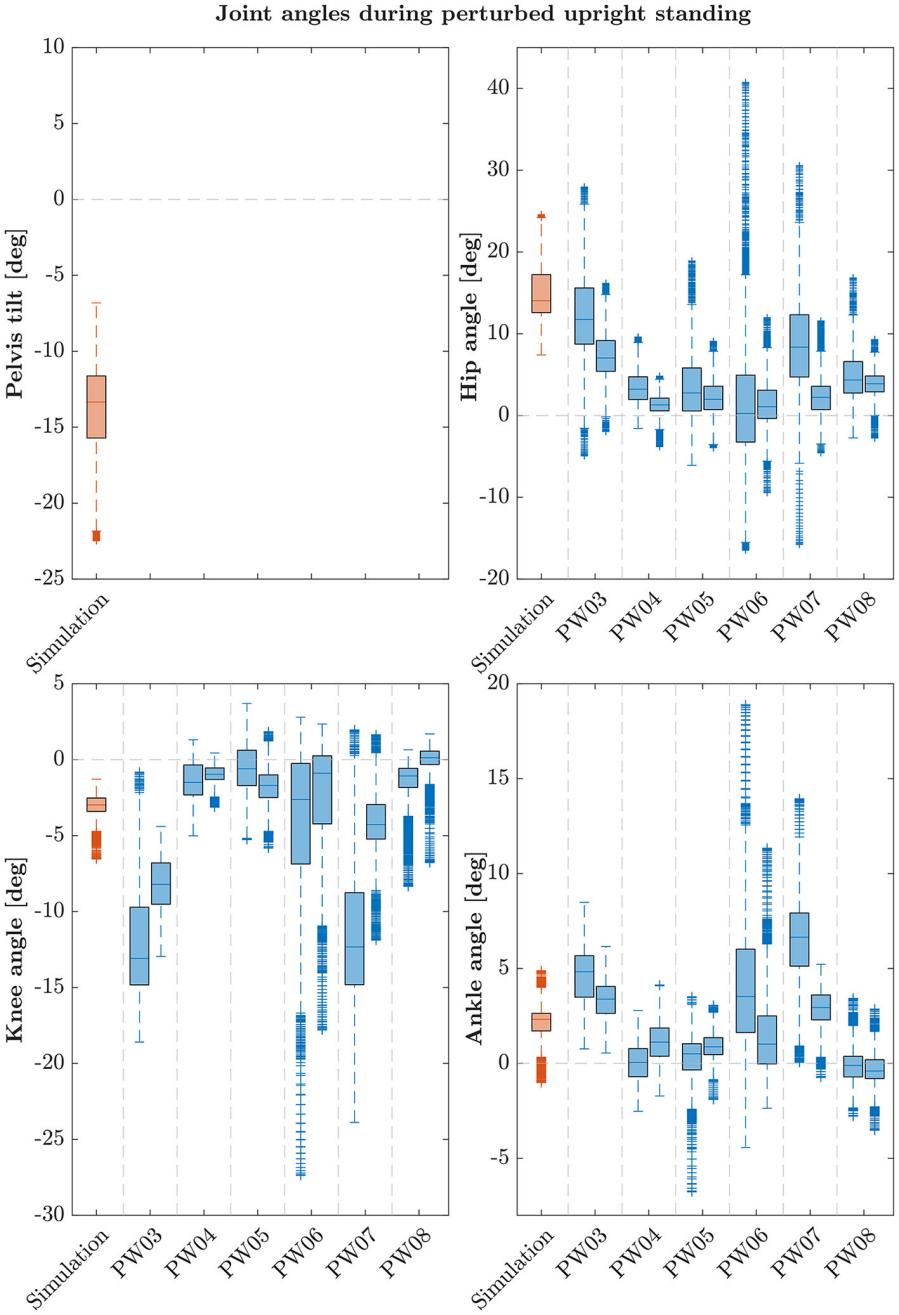
Box-plots showing median and interquartile ranges of joint angles during upright standing on a moving platform of simulation and each participant. During the experiments, each subject fulfilled the perturbed standing task twice. Note that these participants are not the same as in the quiet standing scenario.

**Figure 4:**
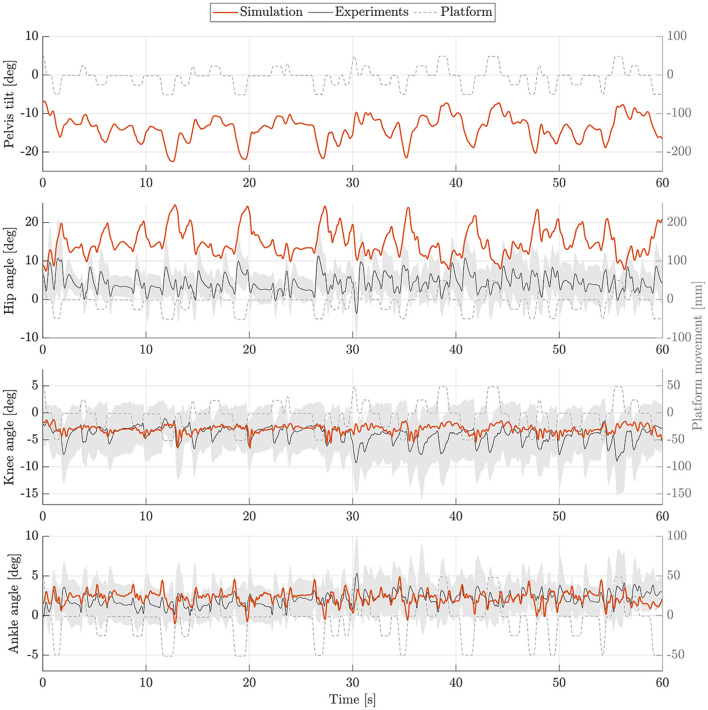
Joint angles during upright standing on a moving platform. Joint angles of simulation (orange) and experiments (mean: black, standard deviation: shaded gray area) are shown for 60 s.

**Figure 5:**
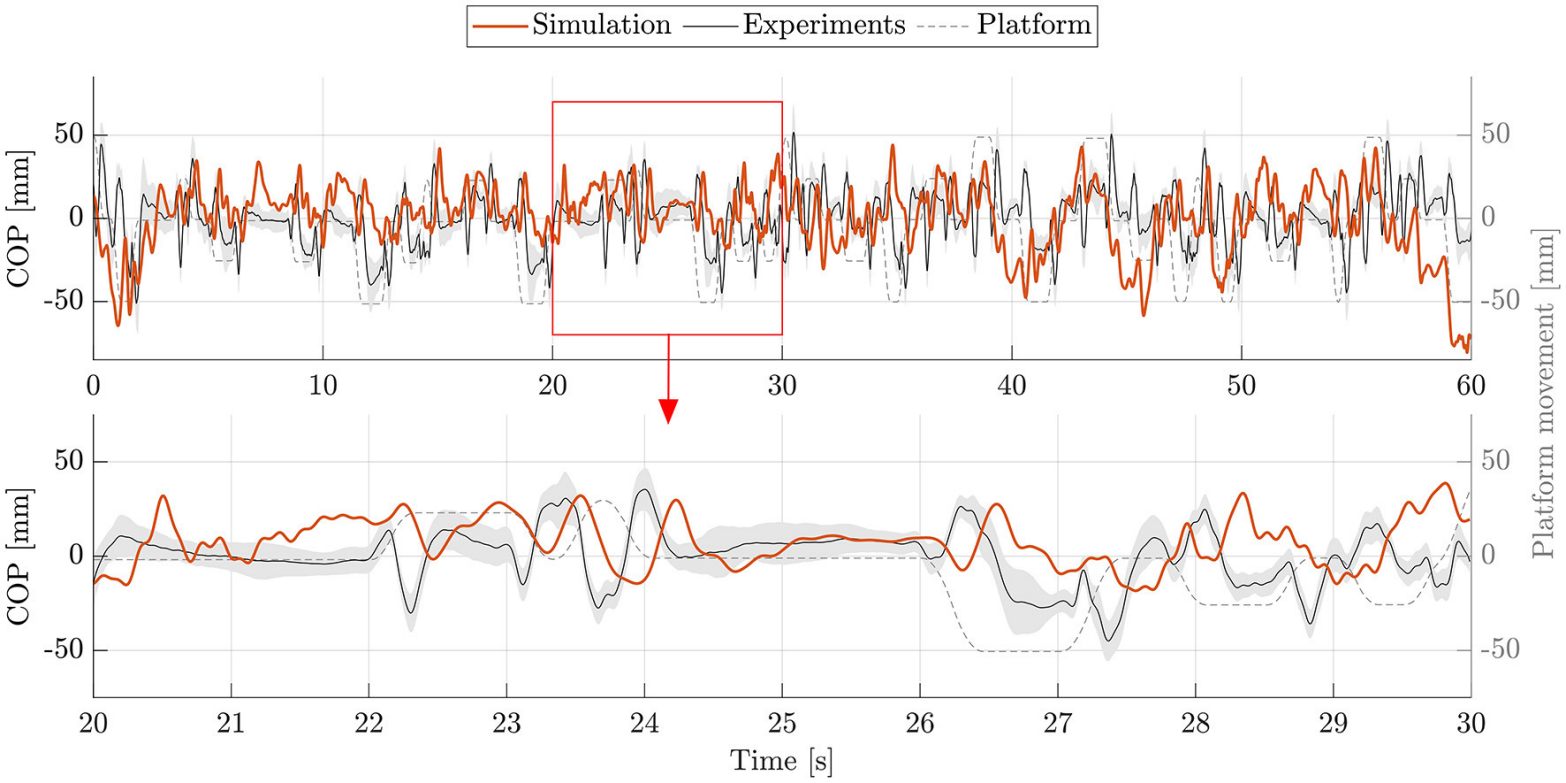
COP during upright standing on a moving platform. The COP of simulation (orange) and experiments (mean: black, standard deviation: shaded gray area) is shown for 60 s. Additionally, an exemplary section of 10 s (highlighted with a red frame) is represented as zoom-in below.

**Figure 6:**
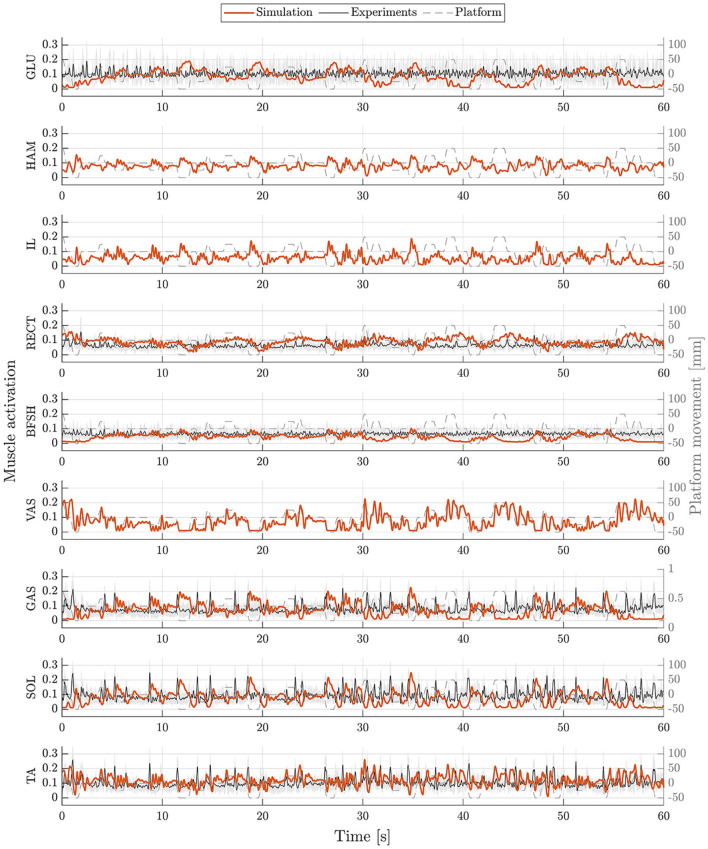
Muscle activations during upright standing on a moving platform. Muscle activations of simulation (orange) and experiments (mean: black, standard deviation: shaded gray area) are represented for 60 s. Muscle activations are shown for gluteus maximus (GLU), hamstrings (HAM), iliopsoas (IL), rectus femoris (RECT), biceps femoris short head (BFSH), vastus intermedius (VAS), gastrocnemius medialis (GAS), soleus (SOL), and tibialis anterior (TA).

**Figure 7:**
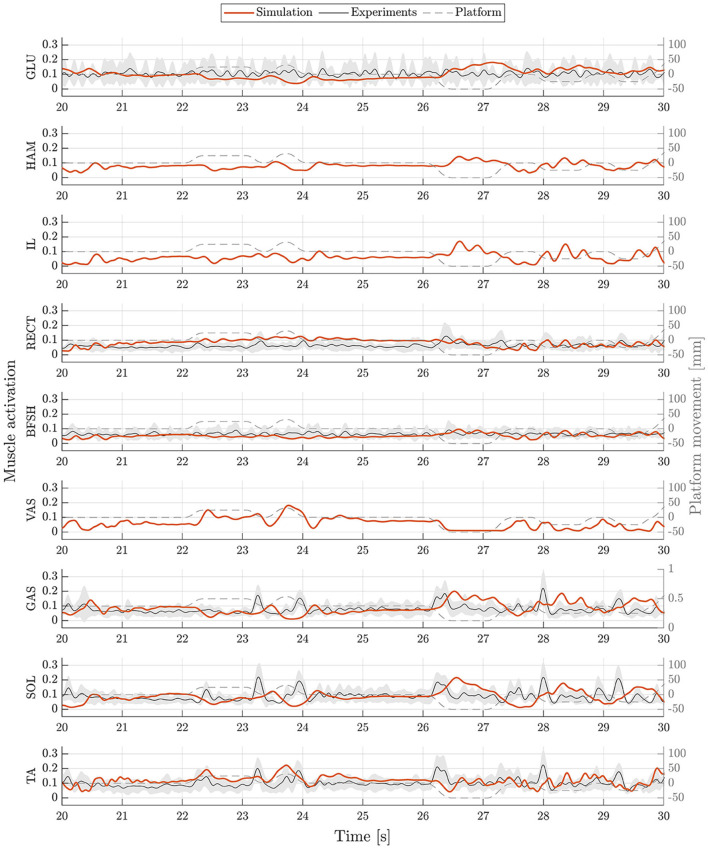
Muscle activations during upright standing on a moving platform. Muscle activations of simulation (orange) and experiments (mean: black, standard deviation: shaded gray area) are represented as an exemplary zoom-in of 10 s. Muscle activations are shown for gluteus maximus (GLU), hamstrings (HAM), iliopsoas (IL), rectus femoris (RECT), biceps femoris short head (BFSH), vastus intermedius (VAS), gastrocnemius medialis (GAS), soleus (SOL), and tibialis anterior (TA).

The postural control model was also capable of maintaining balance on a moving platform. We compared simulation results with the published joint angles (Wang and van den Bogert, 2020). Figure 3 shows joint angles of simulation and each participant of the experiments. As there was no pelvis tilt given in the published dataset, we only compared hip, knee and ankle angles. Resulting joint angle courses of simulation and average experimental data are represented in Figure 4, COP courses in Figure 5. We observed RMSEs of 11.11° (hip angle), 1.86° (knee angle), and 1.04° (ankle angle) between simulation and experiments. Simulation results showed joint angles' ROMs of 8.84° (pelvis tilt), 8.29° (hip angle), 8.73° (knee angle), and 4.16° (ankle angle). Average experimental data showed ROMs of 23.46 ± 14.97° (hip angle), 13.34 ± 8.35° (knee angle), and 9.10 ± 5.47° (ankle angle). The COP range was 124.77 mm during the simulation, 120.43 ± 25.55 mm during experiments. Joint angles' RMSEs, ROMs and ranges of the COP are summarized in Table 3. Additionally, we compared simulated muscle activations with recorded EMG data. In Figures 6, 7, activation courses are represented for 60 s and for 10 s in detail. For the muscles gluteus maximus, rectus femoris, biceps femoris short head, gastrocnemius medialis, soleus and tibialis anterior, we were able to compare simulation results with experimental data. It could be observed, that simulated muscle activations showed comparable behavior in some parts, especially for gastrocnemius medialis, soleus and tibialis anterior, but also differed in several areas from experimental data, especially for gluteus maximus, rectus femoris and biceps femoris short head.

**Table 3:** Joint angles and COP's parameters of the simulation and average experimental data during upright standing on a moving platform.

**Parameters**	**ROM simulation**	**ROM experiments**	**RMSE**
Pelvis tilt (deg)	8.84	N/A	N/A
Hip angle (deg)	8.29	23.46 ± 14.97	11.11
Knee angle (deg)	8.73	13.34 ± 8.35	1.86
Ankle angle (deg)	4.16	9.10 ± 5.47	1.04
COP (mm)	124.77	120.43 ± 25.55	N/A

No pelvis angles were given for experimental results.

The model's resulting optimized parameters, are summarized in Table S1 for the quiet upright standing and Table S2 for the upright standing on a moving platform. Results are given for all free parameters of the neural controller that had been optimized.

## 4 Discussion

In this paper, we aimed to simulate postural control behavior using a musculoskeletal human model and complex sensor feedback of all sensory systems involved in postural control considering physiologically plausible neural delays. Compared to many other postural control models, our model uses all, somatosensory, vestibular as well as visual information for balance control under the influence of neural delays in physiologically plausible ranges. Other models often focus on specific aspects like muscle reflexes based on proprioceptive feedback (Suzuki and Geyer, 2018; Koelewijn and Ijspeert, 2020). Compared to them, we aimed to take into account all sensory information that are considered for postural control by the human body (Peterka, 2018; Jahn and Wühr, 2020). This way, our model covers muscle reflexes based on proprioceptive information and is enhanced by further sensory input. The model is able to fulfill an upright standing task in unperturbed and perturbed situations. Motion behavior shows to be comparable to experimental data of healthy participants for both simulation scenarios. For both, the unperturbed as well as the perturbed situation, the model's motion is comparable to the ones observed during experimental measurements.

We observed RMSEs smaller than 1.9° for ankle and knee angles, only hip and pelvis showed a higher variation between simulation and experiments (4.93–11.11°). This is because the stable standing pose that was found by the optimization, especially during the standing on a moving platform, consists of a higher hip flexion and a more forward leaning torso. As the initial standing pose has influences on the reference control parameters, a different initial pose could improve the torso orientation. Also, the model's reference COP position is currently defined as the midpoint between the two contact spheres of each foot. In reality, the COP might not lie directly in this point. As we could not compare absolute COP positions, simulations and experiments could differ in this aspect. During quiet standing, ROMs are comparable, only the COP range shows to be considerably smaller during the simulation. During the perturbed standing, the simulation's ROM is noticeable smaller for all joint angles, the COP range is comparable to the experiments. It has to be said, that also the experimental data itself showed high standard deviations of the ROMs. This means, that the amount of ROM varies widely between the individual participants. Also, experimental results showed high variation in absolute mean values in general which is shown in Figures 2, 3. We aimed to present a general postural control model, that is able to create physiologically plausible postural control behavior in the first step. The model was not yet personalized and simulations were not provided for different subjects. Therefore, our simulations did not show as much variability as the experimental data. Considering this, simulation results show realistic motion behavior. Besides internal noise of sensory systems and muscle actuators, also low-frequent disturbances caused by breathing or the heart beat influence motion during postural control (Forbes et al., 2018). These effects are currently not considered in our model and could explain the remaining difference between simulation and experimental ROMs. We compared simulated and recorded muscle activations and observed, that muscle activations still varied in several sections, especially for upper leg and hip muscles. Quantitative comparisons are currently not possible, we could only compare muscle activations' time courses. Maximum voluntary contraction measures would be necessary to gain insights into absolute muscle activations during experiments that are extracted via EMG. It has to be mentioned, that not all simulated muscle activations could get compared to experimental data. No EMG data was provided for hamstrings, iliopsoas and vastus intermedius. Since we focused on creating a postural control model that is able to fulfill the previously described tasks based on physiologically plausible assumptions in this first step, muscle activations still seem to vary in some aspects compared to experimental data. In a next step, a more similar muscle activation behavior could be one additional focus aspect.

We used a sagittal plane musculoskeletal human model to simulate postural control. We are aware that not only anterior-posterior, but also medio-lateral movements are relevant in order to holistically simulate motion behavior. Additionally, our current simulation approach creates symmetrical motion. In reality, human motion is never completely symmetrical. Still, a sagittal plane model will already be capable of providing some insights into impaired neural control as well, even if it is not complete. We assumed different neural delays depending on muscle position and sensor information type. It has to be mentioned that the specific amount of different neural delays is still being discussed. In this respect, current models for postural control differ considerably, like with 100 ms (van der Kooij et al., 2001), 120 ms (Jiang et al., 2017), 150 ms (Van Wouwe et al., 2022) or 185 ms (Masani et al., 2006). However, at the same time, postural control models could help to identify neural delays by investigating differences in motion behavior resulting from adaptions of neural delays. Our model assumes a multisensory integration in form of a weighted sum of several sensory information in the feedback loop. Also this internal process of sensory integration of the body is still unclear. Many models use this approach to fuse sensor information (Goodworth and Peterka, 2009; Jiang et al., 2017; Van Wouwe et al., 2022). Other models use for example optimal estimator methods (van der Kooij et al., 2001; Kuo, 2005) to process the information before initiating corresponding model reactions. Some studies inform about frequency-dependencies of the body's sensory systems (Forbes et al., 2018; Peterka, 2018; Jahn and Wühr, 2020). Optimal estimator methods can take this aspect into account. In our approach, this characteristic is currently not addressed. We aimed to simulate the reactive postural control behavior of upright standing. Up to this point, our model considers aspects of spinal and supra-spinal control. Motion aspects such as voluntary movements, are currently not included in the model.

Postural control behavior can differ substantially between subjects. Even in healthy individuals, factors like age influence postural control significantly (Rinaldi et al., 2009; Van Humbeeck et al., 2023). Even though we ensured to include subjects of different sex and age, a higher number of subjects could still influence our reference values. We used experimental data of eight subjects (age: 51.63 ± 23.12 years) to compare them with our simulation results and gain an overall impression of our model compared to human data. In a next step, also aging effects could be considered to model age-specific postural control. Up to this point, our simulations were conducted with a generic OpenSim model. A next step could be to use personalized musculoskeletal human models to get a more subject-specific motion behavior. By using customized models that are adapted to represent specific individuals, even more precise simulation performances and higher similarities would be expected compared to experimental data. To model movements of patients with neural disorders, such as PD, further adaptions to the model are necessary. Depending on the severity and progression of the disease, patients may even exhibit an altered body pose, independent of external perturbations. In addition, the reactive postural control behavior can differ significantly. Therefore, targeted modifications to the model are necessary to achieve accurate simulation results for Parkinson's patients.

Additionally, it is important to keep in mind that even though if a postural control model shows human-like motion behavior, this does not necessarily prove that the model mimics the control processes of real humans. Nevertheless, these models may help to gather insights into the differences between physiological and pathophysiological control.

## 5 Conclusion and outlook

In this paper, we introduced a musculoskeletal postural control model using complex sensor feedback consisting of somatosensory, vestibular and visual information considering physiologically plausible neural delays. It is able to maintain balance in both unperturbed as well as perturbed scenarios. The simulated motion behavior showed to be comparable to empirical data of healthy participants.

This model will serve as a basis to simulate and even characterize motion behavior of persons suffering from neurological disorders like PD. In a next step, parameters that are thought to be the cause of symptoms such as postural control impairments could be adjusted. This will allow us to further investigate the balance behavior of Parkinson's patients and to assess, for example, the effects of different rehabilitation interventions.

## Data availability statement

The raw data supporting the conclusions of this article will be made available by the authors, without undue reservation.

## Ethics statement

The studies involving humans were approved by the Ethics Committee of the FAU Erlangen-Nürnberg. The studies were conducted in accordance with the local legislation, institutional requirements and with the Declaration of Helsinki. The participants provided their written informed consent to participate in this study.

## Author contributions

JS: Conceptualization, Data curation, Formal analysis, Investigation, Methodology, Software, Validation, Visualization, Writing – original draft. SF: Formal analysis, Investigation, Writing – review & editing. IW: Formal analysis, Visualization, Writing – review & editing. HG: Writing – review & editing, Resources. JW: Supervision, Writing – review & editing, Funding acquisition, Resources. BE: Project administration, Supervision, Writing – review & editing, Funding acquisition. AK: Formal analysis, Project administration, Supervision, Writing – review & editing. SW: Funding acquisition, Project administration, Resources, Supervision, Writing – review & editing. JM: Conceptualization, Formal analysis, Funding acquisition, Investigation, Methodology, Project administration, Resources, Supervision, Writing – review & editing.

## References

[B1] Delp S. L. Loan J. P. Hoy M. G. Zajac F. E. Topp E. L. Rosen J. M.. (1990). An interactive graphics-based model of the lower extremity to study orthopaedic surgical procedures. IEEE Trans. Biomed. Eng. 37, 757–67. 10.1109/10.102791 2210784

[B2] Forbes P. A. Chen A. Blouin J.-S. (2018). Sensorimotor control of standing balance. Handb. Clin. Neurol. 159, 61–83. 10.1016/B978-0-444-63916-5.00004-5 30482333

[B3] Geijtenbeek T. (2019). SCONE: open source software for predictive simulation of biological motion. J. Open Source Softw. 4:1421. 10.21105/joss.0142136563640

[B4] Geijtenbeek T. (2021). The Hyfydy Simulation Software. Available online at: https://hyfydy.com

[B5] Goodworth A. D. Peterka R. J. (2009). Contribution of sensorimotor integration to spinal stabilization in humans. J. Neurophysiol. 102, 496–512. 10.1152/jn.00118.2009 19403751 PMC2712260

[B6] Igel C. Hansen N. Roth S. (2007). Covariance matrix adaptation for multi-objective optimization. Evol. Comput. 15, 1–28. 10.1162/evco.2007.15.1.1 17388777

[B7] Jahn K. Wühr M. (2020). “Postural control mechanisms in mammals, including humans,” in The Senses: A Comprehensive Reference, 2nd ed. ed. B. Fritzsch (Oxford: Elsevier), 344–370. 10.1016/B978-0-12-809324-5.24132-1

[B8] Jiang P. Chiba R. Takakusaki K. Ota J. (2017). A postural control model incorporating multisensory inputs for maintaining a musculoskeletal model in a stance posture. Adv. Robot. 31, 55–67. 10.1080/01691864.2016.1266095

[B9] Kaminishi K. Jiang P. Chiba R. Takakusaki K. Ota J. (2019). Postural control of a musculoskeletal model against multidirectional support surface translations. PLoS ONE 14:e0212613. 10.1371/journal.pone.0212613 30840650 PMC6402659

[B10] Koelewijn A. D. Ijspeert A. J. (2020). Exploring the contribution of proprioceptive reflexes to balance control in perturbed standing. Front. Bioeng. Biotechnol. 8:866. 10.3389/fbioe.2020.00866 32984265 PMC7485384

[B11] Kröger S. Watkins B. (2021). Muscle spindle function in healthy and diseased muscle. Skelet. Muscle 11:3. 10.1186/s13395-020-00258-x 33407830 PMC7788844

[B12] Kuo A. D. (2005). An optimal state estimation model of sensory integration in human postural balance. J. Neural Eng. 2, 235–49. 10.1088/1741-2560/2/3/S07 16135887

[B13] Li Y. Levine W. S. Loeb G. E. (2012). A two-joint human posture control model with realistic neural delays. IEEE Trans. Neural Syst. Rehabil. Eng. 20, 738–48. 10.1109/TNSRE.2012.2199333 22692939

[B14] Mahboobin A. Beck C. Moeinzadeh M. Loughlin P. (2002). “Analysis and validation of a human postural control model,” in Proceedings of the 2002 American Control Conference (IEEE Cat. No.CH37301), Vol. 5 (Anchorage, AK: IEEE), 4122–4128. 10.1109/ACC.2002.1024576

[B15] Masani K. Vette A. H. Popovic M. R. (2006). Controlling balance during quiet standing: proportional and derivative controller generates preceding motor command to body sway position observed in experiments. Gait Posture 23, 164–72. 10.1016/j.gaitpost.2005.01.006 16399512

[B16] McManus L. De Vito G. Lowery M. M. (2020). Analysis and biophysics of surface EMG for physiotherapists and kinesiologists: toward a common language with rehabilitation engineers. Front. Neurol. 11:576729. 10.3389/fneur.2020.576729 33178118 PMC7594523

[B17] Millard M. Uchida T. Seth A. Delp S. L. (2013). Flexing computational muscle: modeling and simulation of musculotendon dynamics. J. Biomech. Eng. 135:021005. 10.1115/1.4023390 23445050 PMC3705831

[B18] Peterka R. J. (2018). Sensory integration for human balance control. Handb. Clin. Neurol. 159, 27–42. 10.1016/B978-0-444-63916-5.00002-1 30482320

[B19] Rajagopal A. Dembia C. L. DeMers M. S. Delp D. D. Hicks J. L. Delp S. L.. (2016). Full-body musculoskeletal model for muscle-driven simulation of human gait. IEEE Trans. Biomed. Eng. 63, 2068–2079. 10.1109/TBME.2016.2586891 27392337 PMC5507211

[B20] Rinaldi N. M. Polastri P. F. Barela J. A. (2009). Age-related changes in postural control sensory reweighting. Neurosci. Lett. 467, 225–9. 10.1016/j.neulet.2009.10.042 19840830

[B21] Selinger J. C. O'Connor S. M. Wong J. D. Donelan J. M. (2015). Humans can continuously optimize energetic cost during walking. Curr. Biol. 25, 2452–2456. 10.1016/j.cub.2015.08.016 26365256

[B22] Seth A. Hicks J. L. Uchida T. K. Habib A. Dembia C. L. Dunne J. J.. (2018). OpenSim: simulating musculoskeletal dynamics and neuromuscular control to study human and animal movement. PLoS Comput. Biol. 14:1006223. 10.1371/journal.pcbi.1006223 30048444 PMC6061994

[B23] Shanbhag J. Wolf A. Wechsler I. Fleischmann S. Winkler J. Leyendecker S.. (2023). Methods for integrating postural control into biomechanical human simulations: a systematic review. J. Neuroeng. Rehabil. 20:111. 10.1186/s12984-023-01235-3 37605197 PMC10440942

[B24] Suzuki Y. Geyer H. (2018). “A neuro-musculo-skeletal model of human standing combining muscle-reflex control and virtual model control,” in Annual International Conference of the IEEE Engineering in Medicine and Biology Society. IEEE Engineering in Medicine and Biology Society. Annual International Conference 2018 (Honolulu, HI: IEEE), 5590–5593. 10.1109/EMBC.2018.8513543 30441603

[B25] van der Kooij H. Jacobs R. Koopman B. van der Helm F. (2001). An adaptive model of sensory integration in a dynamic environment applied to human stance control. Biol. Cybern. 84, 103–115. 10.1007/s004220000196 11205347

[B26] Van Humbeeck N. Kliegl R. Krampe R. T. (2023). Lifespan changes in postural control. Sci. Rep. 13:541. 10.1038/s41598-022-26934-0 36631521 PMC9834247

[B27] Van Wouwe T. Ting L. H. Groote F. (2022). An approximate stochastic optimal control framework to simulate nonlinear neuro-musculoskeletal models in the presence of noise. PLoS Comput. Biol. 18:1009338. 10.1371/journal.pcbi.1009338 35675227 PMC9176817

[B28] Versteeg C. S. Ting L. H. Allen J. L. (2016). Hip and ankle responses for reactive balance emerge from varying priorities to reduce effort and kinematic excursion: a simulation study. J. Biomech. 49, 3230–3237. 10.1016/j.jbiomech.2016.08.007 27543251 PMC5074864

[B29] Vicon Motion Systems Limited UK (2021). Vicon Plug-in Gait Reference Guide. Oxford: Vicon Motion Systems Ltd UK.

[B30] Wang H. van den Bogert A. (2020). Standing Balance Experiment with Long Duration Random Pulses Perturbation. Zenodo. 10.5281/zenodo.3819630

[B31] Welch T. D. J. Ting L. H. (2008). A feedback model reproduces muscle activity during human postural responses to support-surface translations. J. Neurophysiol. 99, 1032–1038. 10.1152/jn.01110.2007 18094102

[B32] Werling K. Bianco N. A. Raitor M. Stingel J. Hicks J. L. Collins S. H.. (2023). AddBiomechanics: automating model scaling, inverse kinematics, and inverse dynamics from human motion data through sequential optimization. PLoS ONE 18:e0295152. 10.1371/journal.pone.0295152 38033114 PMC10688959

[B33] Winter D. (1995). Human balance and posture control during standing and walking. Gait Posture 3, 193–214. 10.1016/0966-6362(96)82849-9

[B34] Yin K. Chen J. Xiang K. Pang M. Tang B. Li J.. (2020). Artificial human balance control by calf muscle activation modelling. IEEE Access 8, 86732–86744. 10.1109/ACCESS.2020.2992567

