# Supplementary material for: A sensorimotor enhanced neuromusculoskeletal model for simulating postural control of upright standing
Source: Front Neurosci. 2024 May 15;18:1393749. doi: 10.3389/fnins.2024.1393749 (PMC11133552; doi:10.3389/fnins.2024.1393749)
Supplement: Supplementary file 1 [file Data_Sheet_1.pdf]

Supplementary Material

Table S1. Optimized model's control parameters for quiet upright standing.

Parameter	Muscle	Value	Parameter	Muscle	Value
u_0	Gluteus maximus	0.0320	$K_{\ddot{x}}$	Gluteus maximus	-0.0215
	Hamstrings	0.0026		Hamstrings	0.0988
	Iliopsoas	0.0130		Iliopsoas	0.1508
	Rectus femoris	0.0010		Rectus femoris	-0.0482
	Biceps femoris short head	0.0381		Biceps femoris short head	0.0017
	Vastus intermedius	0.0824		Vastus intermedius	0.1089
	Gastrocnemius medialis	0.0012		Gastrocnemius medialis	0.0739
	Soleus	0.0011		Soleus	0.0935
	Tibialis anterior	0.0080		Tibialis anterior	-0.1366
$l_{m,0}$	Gluteus maximus	1.7927	K_{φ}	Gluteus maximus	-0.0409
	Hamstrings	0.9323		Hamstrings	0.1689
	Iliopsoas	0.9938		Iliopsoas	0.0055
	Rectus femoris	1.1451		Rectus femoris	0.2577
	Biceps femoris short head	1.6881		Biceps femoris short head	0.6400
	Vastus intermedius	1.9113		Vastus intermedius	0.4635
	Gastrocnemius medialis	1.5572		Gastrocnemius medialis	-0.1574
	Soleus	1.2838		Soleus	0.2280
	Tibialis anterior	0.8191		Tibialis anterior	0.3322
K_l	Gluteus maximus	0.0922	$K_{\ddot{\varphi}}$	Gluteus maximus	-0.0218
	Hamstrings	1.1476		Hamstrings	0.0324
	Iliopsoas	0.9095		Iliopsoas	0.0773
	Rectus femoris	-0.2205		Rectus femoris	-0.0343
	Biceps femoris short head	1.8251		Biceps femoris short head	0.1611
	Vastus intermedius	1.8043		Vastus intermedius	0.1415
	Gastrocnemius medialis	-0.1301		Gastrocnemius medialis	0.0286
	Soleus	0.7049		Soleus	0.0224
	Tibialis anterior	2.3950		Tibialis anterior	0.1395
$K_{\dot{l}}$	Gluteus maximus	0.0855	K_x	Gluteus maximus	0.5562
	Hamstrings	0.0967		Hamstrings	0.2289
	Iliopsoas	0.0255		Iliopsoas	0.6433
	Rectus femoris	0.1035		Rectus femoris	-1.1930
	Biceps femoris short head	0.0179		Biceps femoris short head	-0.9791
	Vastus intermedius	0.0918		Vastus intermedius	-1.0271
	Gastrocnemius medialis	-0.0799		Gastrocnemius medialis	1.5026
	Soleus	0.1224		Soleus	2.8035
	Tibialis anterior	0.1117		Tibialis anterior	0.1921
K_F	Gluteus maximus	0.4586	$K_{\dot{x}}$	Gluteus maximus	0.2127
	Hamstrings	0.1638		Hamstrings	0.4805
	Iliopsoas	-0.3334		Iliopsoas	0.2980
	Rectus femoris	2.1993		Rectus femoris	-0.0374
	Biceps femoris short head	-0.2075		Biceps femoris short head	0.4534
	Vastus intermedius	0.3506		Vastus intermedius	-0.0913
	Gastrocnemius medialis	0.1121		Gastrocnemius medialis	0.7881
	Soleus	0.1368		Soleus	0.9074
	Tibialis anterior	0.1287		Tibialis anterior	0.3902
K_{cop}	Gluteus maximus	0.1407			
	Hamstrings	0.3716			
	Iliopsoas	0.2863			
	Rectus femoris	0.3203			
	Biceps femoris short head	0.3746			
	Vastus intermedius	0.2360			
	Gastrocnemius medialis	0.3777			
	Soleus	0.2443			
	Tibialis anterior	0.4654			

Table S2. Optimized model's control parameters for upright standing on a moving platform.

Parameter	Muscle	Value	Parameter	Muscle	Value
u_0	Gluteus maximus	0.0033	$K_{\ddot{x}}$	Gluteus maximus	0.0335
	Hamstrings	0.0234		Hamstrings	0.0745
	Iliopsoas	0.0091		Iliopsoas	0.0973
	Rectus femoris	0.1334		Rectus femoris	0.1327
	Biceps femoris short head	0.0014		Biceps femoris short head	0.0879
	Vastus intermedius	0.0363		Vastus intermedius	0.0346
	Gastrocnemius medialis	0.0012		Gastrocnemius medialis	0.1431
	Soleus	0.0014		Soleus	0.1305
	Tibialis anterior	0.0603		Tibialis anterior	0.0471
$l_{m,0}$	Gluteus maximus	0.7867	K_{φ}	Gluteus maximus	0.2862
	Hamstrings	0.9118		Hamstrings	0.3570
	Iliopsoas	0.9880		Iliopsoas	0.2498
	Rectus femoris	1.4404		Rectus femoris	0.5601
	Biceps femoris short head	1.6027		Biceps femoris short head	0.3247
	Vastus intermedius	0.5764		Vastus intermedius	0.6088
	Gastrocnemius medialis	1.5788		Gastrocnemius medialis	0.1085
	Soleus	1.6233		Soleus	0.3217
	Tibialis anterior	0.8104		Tibialis anterior	0.6897
K_l	Gluteus maximus	1.0849	$K_{\ddot{\varphi}}$	Gluteus maximus	0.0009
	Hamstrings	0.4791		Hamstrings	0.0394
	Iliopsoas	1.4195		Iliopsoas	0.0580
	Rectus femoris	0.1850		Rectus femoris	0.0721
	Biceps femoris short head	0.8555		Biceps femoris short head	0.0360
	Vastus intermedius	2.1029		Vastus intermedius	0.0541
	Gastrocnemius medialis	2.5657		Gastrocnemius medialis	0.0299
	Soleus	1.6105		Soleus	0.0348
	Tibialis anterior	0.7319		Tibialis anterior	0.0703
$K_{\dot{l}}$	Gluteus maximus	-0.0012	K_x	Gluteus maximus	0.9356
	Hamstrings	0.0443		Hamstrings	0.3523
	Iliopsoas	-0.0012		Iliopsoas	0.7601
	Rectus femoris	0.1532		Rectus femoris	0.0345
	Biceps femoris short head	0.0845		Biceps femoris short head	0.9610
	Vastus intermedius	0.0433		Vastus intermedius	0.0256
	Gastrocnemius medialis	0.0025		Gastrocnemius medialis	1.9208
	Soleus	0.0703		Soleus	1.1598
	Tibialis anterior	0.1890		Tibialis anterior	0.7472
K_F	Gluteus maximus	0.6166	$K_{\dot{x}}$	Gluteus maximus	0.1310
	Hamstrings	0.3637		Hamstrings	0.3909
	Iliopsoas	0.3828		Iliopsoas	0.4822
	Rectus femoris	0.1149		Rectus femoris	-0.0105
	Biceps femoris short head	0.1933		Biceps femoris short head	0.0765
	Vastus intermedius	1.5825		Vastus intermedius	-0.2015
	Gastrocnemius medialis	-0.3130		Gastrocnemius medialis	0.8157
	Soleus	0.4613		Soleus	0.7274
	Tibialis anterior	0.5641		Tibialis anterior	0.1289
K_{cop}	Gluteus maximus	0.0507			
	Hamstrings	0.0529			
	Iliopsoas	0.2882			
	Rectus femoris	0.4073			
	Biceps femoris short head	0.3965			
	Vastus intermedius	0.0950			
	Gastrocnemius medialis	-0.0109			
	Soleus	-0.0352			
	Tibialis anterior	0.0949			
